# Association of a Left Atrial Diverticulum with Adverse Events During Catheter Ablation for Atrial Fibrillation

**DOI:** 10.3390/jcm14093041

**Published:** 2025-04-28

**Authors:** Koki Yamaoka, Seiji Takatsuki, Shuhei Yano, Yukihiro Himeno, Shuhei Yamashita, Susumu Ibe, Takahiko Nishiyama, Yoshinori Katsumata, Takehiro Kimura, Masaki Ieda

**Affiliations:** Department of Cardiology, Keio University School of Medicine, Shinanomachi 35, Shinjuku-ku, Tokyo 160-8582, Japan

**Keywords:** left atrial diverticulum, catheter ablation, atrial fibrillation, atrial tachycardia, complications, cardiac computed tomography

## Abstract

**Background/Objectives:** Left atrial diverticula (LADs) have been reported to potentially be associated with arrhythmic substrates, thromboembolic events, and complications during catheter ablation for atrial fibrillation (AF), but their clinical significance remains unclear. This study aimed to assess the prevalence, location, and potential relationship with complications during AF catheter ablation using preoperative CT. **Methods:** This study included 595 consecutive patients undergoing AF catheter ablation at Keio University Hospital from April 2021 to February 2024. Preoperative ECG-gated cardiac MDCT scans were analyzed to assess the presence and location of the LAD. Intraoperative adverse events were documented, and the association between the LAD and mechanical complications, such as a cardiac perforation and tamponade, was evaluated. **Results:** A total of 595 patients undergoing catheter ablation for AF or atrial tachycardia (AT) were included, with 210 (35.3%) found to have an LAD. No significant differences in age, sex, body mass index, or arrhythmia type were observed between patients with or without an LAD. LADs were most commonly located in the anterior region of the right superior pulmonary vein (53.4% of cases), followed by the anterior region of the left superior pulmonary vein (15% of cases). Perioperative complications occurred in 12 cases (2.0%), with 7 in the LAD group and 5 in the non-LAD group. Mechanical complications were observed exclusively in the LAD group (*n* = 4), with three of the cases associated with LADs. In all cases, LADs were present in the anterior region of the right superior pulmonary vein and were caused by the accidental insertion of an angiographic catheter into the LAD during pulmonary venography before insertion of the ablation catheter into the left atrium. However, all cases were hemodynamically stable, and the procedures were completed as planned. **Conclusions:** LADs are a more common anatomical structure than generally recognized and may be associated with mechanical complications during AF catheter ablation. Identifying the presence of an LAD on preoperative CT is crucial for predicting potential risks.

## 1. Introduction

Preoperative cardiac CT is usually performed for an anatomical and intraatrial thrombus evaluation before catheter ablation for atrial fibrillation (AF), and a left atrial diverticulum (LAD) is occasionally observed. Similar structures include an atrial accessory appendage and an atrial aneurysm, defined by their origin, contractility, and morphology. LADs originate from a common pulmonary vein, exhibit contractility, and have a sac-like structure with a smooth or trabeculated surface, circumscriptive ostium, and narrow neck [1]. Pathology reports show that the wall of the LAD is significantly thinner than that of the surrounding left atrial myocardium [2]. Clinically, LADs are considered one of the most interesting structures, as they have been suggested to be associated with thrombus and cerebrovascular events [3,4,5]. Numerous studies have reported potential associations between LADs and AF. Most studies indicated no difference in the prevalence of an LAD between sinus rhythm and AF [1,6,7,8]. Additionally, many reports have suggested that there is no significant difference in the recurrence rates after AF catheter ablation regardless of the presence of an LAD [9,10]. However, from an electrophysiological perspective, it has been reported that the local potentials within the LAD are more fractionated and exhibit a higher impedance than the surrounding left atrial myocardium [11]. Moreover, there are cases in which premature atrial contractions originating from the LAD have been reported as AF triggers, indicating that LADs could potentially serve as an arrhythmogenic substrate [12]. Based on the morphological characteristics and common locations of LADs, there have been previous reports of the potential risk of complications, such as cardiac perforations and stem pops due to catheter trapping, during AF catheter ablation that requires manipulation within the left atrium [2,13,14]. This information is extremely important for safely performing catheter ablation, but few reports have examined practical case reports and the relationship between the LAD and procedure-related adverse events.

This study aimed to thoroughly evaluate the association between the LAD and complications during AF catheter ablation. We reassessed the prevalence and morphological characteristics of LADs in patients undergoing AF catheter ablation and examined their association with procedure-related complications.

## 2. Materials and Methods

### 2.1. Study Design and Patient Population

In this study, we included 595 consecutive patients undergoing AF catheter ablation at a single center (Keio University Hospital) from April 2021 to February 2024. We retrospectively analyzed preoperative ECG-gated cardiac MDCT scans to assess the presence and location of the LADs. Furthermore, we examined the occurrence of intraoperative complications during ablation in each patient and evaluated the association between the LAD and those complications. The arrhythmias treated included paroxysmal AF, persistent AF, recurrent AF, and atrial tachycardia (AT), with procedures involving intra-left atrial manipulation. Patients were excluded if CT image quality was suboptimal, if three-dimensional reconstruction was not feasible, or if the administration of contrast agents was contraindicated. Although strict exclusion criteria were not predefined, clearly non-diagnostic CT images with severely limited quality that precluded accurate anatomical evaluation were excluded from the analysis. The patient characteristics of our study population are listed in Table 1.

This retrospective observational study was approved by the Institutional Review Board of Keio University Hospital (Approval No. 20200331, approved on 30 March 2021) and conducted in accordance with the Declaration of Helsinki. Written informed consent was obtained for catheter ablation procedures and contrast-enhanced CT scans. Patient data were collected using an opt-out approach for analysis.

### 2.2. Cardiac CT Protocol

ECG-gated cardiac multidetector computed tomography (MDCT) scans were performed in 595 AF patients within a timeframe ranging from one day to two weeks before undergoing AF catheter ablation. The majority of the patients underwent CT scans at our hospital using scanners from Canon Medical Systems or GE HealthCare. However, some patients were referred from other hospitals, where a portion of those patients underwent CT scans prior to referral. Scanning was conducted in the cranio-caudal direction using a retrospective ECG-gated mode.

At our hospital, cardiac CT imaging was carried out using either a 320-detector row CT scanner (Aquilion ONE GENESIS Edition, Canon Medical Systems, Ōtawara, Tochigi Prefecture) or a 256-detector row CT scanner (Revolution Apex, GE HealthCare, Chicago, IL, USA). Scanning was performed in the cranio-caudal direction using a 160 mm volume scan and a prospective ECG-gated mode.

For the 320-detector row scanner, the scanning parameters included a gantry rotation time of 275 ms, collimation width of 320 × 0.5 mm, and tube voltage of 100 kVp, with the tube current controlled via an automatic exposure control (AEC) system. Similarly, the 256-detector row scanner was operated with a gantry rotation time of 234 ms, collimation width of 256 × 0.625 mm, and tube voltage of 100 kVp, with the tube current also regulated by AEC.

A non-ionic contrast agent with an iodine concentration of 350–370 mg/mL was administered at a fractional dose of 25 mg/kg/s over 12 s, followed by a 20 mL saline flush at the same flow rate. Bolus tracking was performed with the region of interest (ROI) set in the left atrium. Image acquisition was automatically triggered when the contrast enhancement reached a predefined threshold of 150 Hounsfield units (HU).

Image reconstruction protocols were tailored to the scanner type. For the 320-detector row scanner, reconstructed images featured a slice width of 0.5 mm and a slice interval of 0.25 mm, yielding a total of 640 slices. For the 256-detector row scanner, reconstructed images had a slice width and interval of 0.625 mm, producing 256 slices.

### 2.3. Image Analysis

In general, LADs are considered cyst-like structures protruding from the atrial cavity and have been defined based on embryological and morphological characteristics in previous studies, as mentioned above. However, some LADs do not appear as typical cysts. Therefore, in this study, we defined LADs as structures protruding outward from the plane of the left atrial wall, regardless of etiology, based on previous morphological descriptions. For all patients, the presence, number, and location of LADs were recorded. To differentiate LADs from morphologically similar structures such as accessory atrial appendages or left atrial aneurysms, each finding was independently reviewed by two experienced cardiologists involved in catheter ablation, considering anatomical features, location, and the absence of communication with the pulmonary veins. If the diagnosis of LAD was uncertain, they additionally reviewed multiple CT planes, including axial and coronal views, in addition to three-dimensional reconstructed images.

The prevalence and location of the LAD in all patients were investigated. The location of the LAD was characterized on the basis of the atrial wall and classified into 16 segments: right superior, left superior, right inferior, left inferior, right upper posterior, right lower posterior, left upper posterior, left lower posterior, right upper anterior, right lower anterior, left upper anterior, left lower anterior, upper septum, lower septum, upper lateral, and lower lateral. The number and percentage of each segment are illustrated in the figure.

### 2.4. Association of the LAD with Intraoperative Complications

Intraoperative complications were examined from the ablation records of all patients. Cardiac perforations and cardiac tamponades were considered as potential complications related to LADs, and those were collectively defined as mechanical complications. The number of complications, particularly mechanical complications, was compared between the patients with or without LADs. Thereafter, we reviewed the intraoperative fluoro-scopic images and 3D mapping systems in cases with mechanical complications, identifying those complications clearly caused by LADs.

### 2.5. Statistical Analysis

Continuous data are expressed as the mean ± SD or median IQR, and categorical variables are presented as percentages. Comparisons of continuous variables between the two groups, classified based on the presence or absence of LAD, were conducted using the independent t-test for normally distributed data and the Mann–Whitney U test for non-normally distributed data. Categorical variables were compared using Fisher’s exact test or the chi-square test, as appropriate. Descriptive statistics were calculated. The prevalence and distribution of the LADs are expressed as the number of patients and percentages, respectively.

The association between the presence of LADs and complications was assessed using Fisher’s exact test. In view of the very low number of mechanical complications, Firth’s penalized logistic regression was additionally applied when complete separation was suspected, in place of conventional logistic regression, both in univariate and in multivariate analyses, adjusted for age. A two-tailed probability value of 0.05 indicated a statistically significant result. All statistical analyses were performed using dedicated EZR software (Version 1.61)

## 3. Results

### 3.1. Prevalence of LADs

A total of 595 patients who underwent catheter ablation of AF or AT and had pre-procedural CT imaging were included in the study. Detailed patient characteristics are summarized in Table 1. Among those patients, 210 (35.3%) were found to have LADs.

There were no significant differences between the patients with or without LADs in terms of their age (LAD present: 65.6 ± 10.3 years vs. LAD absent: 64.2 ± 10.2 years, *p* = 0.1), sex (female: 19.0% vs. 14.8%, *p* = 0.2), or body mass index (24.4 ± 3.7 kg/m^2^ vs. 24.6 ± 4.0 kg/m^2^, *p* = 0.6). Additionally, no significant differences were observed in the type of arrhythmia targeted for ablation, CHADS_2_ or CHA_2_DS_2_-VASc scores, or the prevalence of comorbidities.

Regarding the laboratory and imaging findings, there were no significant differences in the brain natriuretic peptide (BNP) levels (82.9 pg/mL [33.1, 158.5] vs. 67.3 pg/mL [38.0, 157.1], *p* = 1.0), left ventricular end-diastolic diameter (LVEDd) (4.7 cm [4.4, 5.2] vs. 4.7 cm [4.3, 5.1], *p* = 0.5), left ventricular ejection fraction (LVEF) (61.1% [56.1, 65.0] vs. 62.1% [55.4, 65.3], *p* = 0.8), or left atrial diameter (4.0 cm [3.6, 4.5] vs. 4.1 cm [3.7, 4.6], *p* = 0.2) between the two groups. However, although still within the normal range and of unclear clinical significance, the E/e’ ratio of the interventricular septum was significantly higher in the LAD group (9.1 [0.4, 33.2] vs. 10.4 [4.7, 33.2], *p* = 0.01).

### 3.2. Location and Frequency of the LADs

**Figure 1:**
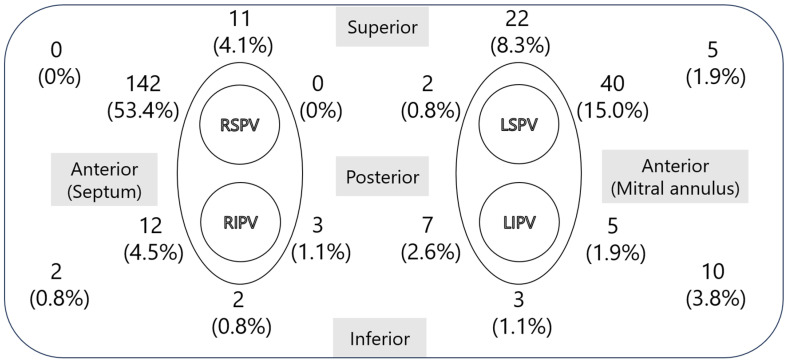
Locations and frequencies of LADs. This figure shows the distribution of LADs in the left atrium in 595 cases. The left side of the figure represents the medial left atrium, and the top represents the superior aspect. The most common location was the anterosuperior aspect of the septum, on the anterior side of the right superior pulmonary vein, followed by the anterior side of the left superior pulmonary vein. LAD, left atrial diverticulum; RSPV, right superior pulmonary vein; RIPV, right inferior pulmonary vein; LSPV, left superior pulmonary vein; LIPV, left inferior pulmonary vein.

The locations and frequencies of the LADs are presented in Figure 1. LADs were identified at various sites within the left atrium, with the anterosuperior aspect of the septum or anterior region of the right superior pulmonary vein representing the most common locations, accounting for 53.4% of the cases across the 16 segments. The anterior region of the left superior pulmonary vein was the second most frequent site, observed in 15% of cases.

### 3.3. Intraoperative Complications

Perioperative complications occurred in 12 of 595 cases (2.0%). Complications occurred in 12 cases: 7 in the LAD group and 5 in the non-LAD group. Cardiac perforations (*n* = 3), cardiac tamponades (*n* = 1), myocardial infarctions (*n* = 1), and arterio-venous shunts (*n* = 1) were observed only in the LAD group, while a cerebral infarction (*n* = 1) and esophageal ulcer (*n* = 1) occurred only in the non-LAD group. Phrenic nerve injury was reported in both groups (LAD group: 1, non-LAD group: 3). Mechanical complications, defined as cardiac perforations and cardiac tamponades, were observed in four cases. All four occurred in the LAD group and were not observed in the non-LAD group (*p* = 0.02) (Table 2). Due to the very low incidence of mechanical complications and the presence of complete separation in the dataset, as mechanical complications occurred only in the LAD group, Firth’s penalized logistic regression was used to reduce bias associated with rare events. The results are shown in Table 3. Consistent with Fisher’s exact test, the presence of an LAD was significantly associated with mechanical complications (odds ratio: 16.8; 95% CI: 1.78–2230.52; *p* = 0.01). In the multivariate analysis adjusted for age, the association remained significant (odds ratio: 17.82; 95% CI: 1.88–2369.95; *p* < 0.01).

**Figure 2:**
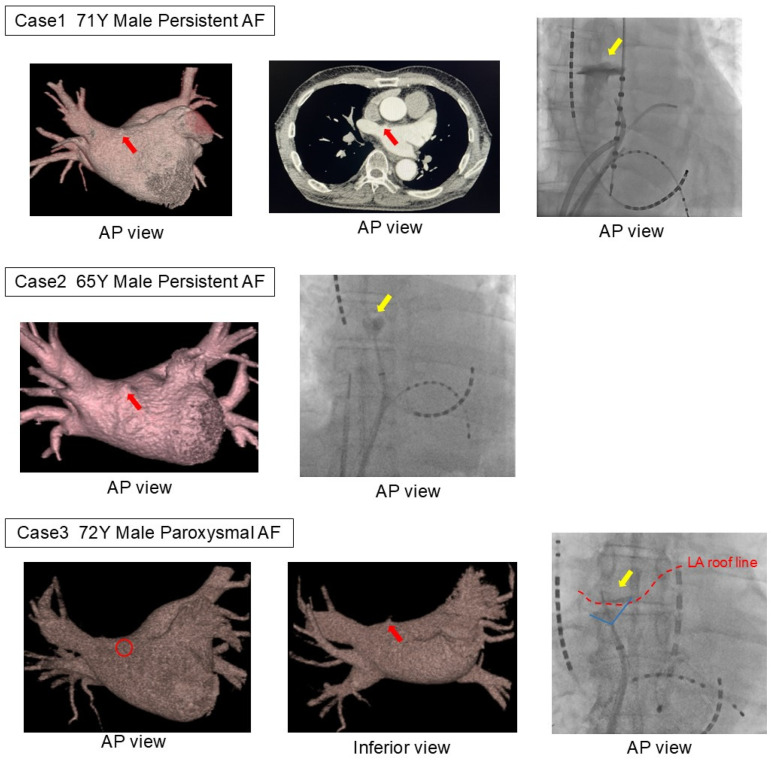
Three patients with LAD-related complications. In all three cases, extravasation was observed during pulmonary vein angiography using an angioburman catheter. In all cases, an LAD was found on the anterior side of the right superior pulmonary vein, the common site of the LAD. The catheter had inadvertently entered the diverticulum during contrast injection immediately after transitioning from angiography of the left pulmonary vein to that of the right pulmonary vein. The red arrow indicates the left atrial diverticulum on the CT image, the yellow arrow points to the extravasation of contrast observed in the fluoroscopic image, and the red dashed line represents the roof line of the left atrium. LA, left atrium; LAD, left atrial diverticulum.

Among the mechanical complications in the LAD group, three cases were cardiac perforations caused by LADs, while the remaining case was a cardiac tamponade due to a steam pop during the creation of the cavo-tricuspid isthmus block line. All LAD-related mechanical complications occurred during the first AF catheter ablation, with the LAD consistently localized in the anterior region of the right superior pulmonary vein (Figure 2).

The first case involved a 71-year-old male undergoing his initial ablation of persistent AF, with a planned BOX isolation using radiofrequency catheter ablation. Pulmonary venography was first performed in the left pulmonary vein using an 8.5Fr long sheath and a 5F angiographic catheter before advancing the ablation catheter into the left atrium. While rotating the sheath clockwise to insert the catheter into the right superior pulmonary vein, the catheter tip inadvertently entered the LAD, causing contrast extravasation. As hemodynamic stability was maintained without a rapid pericardial effusion accumulation, the procedure was continued. Due to the risk of catheter entrapment, radiofrequency ablation was switched to cryoballoon ablation. The contrast pooling resolved relatively early during the procedure. The second case involved a 65-year-old male undergoing his first ablation of persistent AF, with a planned cryoballoon ablation. Similar to the first case, pulmonary venography was performed using an 8.5Fr long sheath and a 5F angiographic catheter. While rotating the sheath clockwise to insert the catheter into the right superior pulmonary vein, contrast extravasation from the diverticulum was observed. Hemodynamic stability was maintained, and the procedure was successfully completed as planned. The third case involved a 72-year-old male undergoing his first ablation for paroxysmal AF, also with a planned cryoballoon ablation. As in the previous cases, contrast extravasation from the diverticulum occurred at the same procedural stage. Hemodynamic stability was maintained, and the procedure proceeded as planned.

## 4. Discussion

Previous meta-analyses in diagnostic radiology have reported a prevalence of LADs of 29.8%, with a notably higher prevalence of 40.6% among Asians [15]. Additionally, 78.7% of LAD cases were identified in the anterosuperior aspect of the left atrium. In this study, which involved a cohort of Asian patients undergoing catheter ablation of atrial arrhythmias, the prevalence of LADs was found to be 35.3%, with a similar predilection for the anterosuperior region.

Several factors may explain why the presence of an LAD increases the risk of complications during catheter ablation. First, the tips of various catheters used during the procedure may become physically caught on the LAD, potentially causing damage. The average size of the LAD has been reported as 5.3 ± 2.9 × 5.6 ± 3.3 mm [2], which corresponds to a dimension that could allow the tips of various catheters to become lodged. Additionally, the wall thickness of the LAD is reported to be significantly thinner than that of the surrounding left atrium (0.89 ± 0.46 mm vs. 2.39 ± 0.83 mm) [2], which may elevate the risk of cardiac perforation. Second, delivering energy with an ablation catheter near the LAD may increase the risk of steam pops. Steam pops are known to result from tissue overheating and are particularly prone to occur in areas with thinner myocardial walls [16]. Additionally, when a catheter becomes wedged within a diverticulum, inadequate irrigation and reduced blood flow may further elevate the risk of steam pops. Lastly, the anterior aspect of the right superior pulmonary vein, a common location for LADs, is frequently a primary target during pulmonary vein isolation. In this area, the ablation catheter tends to come into contact with the atrial wall at a perpendicular angle. Increased catheter manipulation required in this region may further elevate the risk of complications in patients with LADs.

All three patients with complications reported in this study had LADs in the anterior region of the right superior pulmonary vein, which is the typical predilection site. In all cases, extravasation was observed during pulmonary venography performed prior to the pulmonary vein isolation with a 5F angiographic catheter, wedged into the LAD. Considering the anatomical relationship from the septal puncture point, catheter manipulation around the anterior region of the right superior pulmonary vein, the most common site for an LAD, is more likely to result in mechanical complications, as the catheter comes into perpendicular contact with the LAD.

A previous case series has reported complications attributable to LADs [13]. Among five cases of cardiac tamponade during AF catheter ablation, an LAD was identified in four patients. Notably, in two out of three cases requiring surgical intervention, the source of bleeding was confirmed to be the LAD. By contrast, in all three cases in this study, hemodynamic collapse did not occur after extravasation, and there was no sudden increase in the pericardial fluid accumulation, so the procedure, including the pulmonary vein isolation, was completed as planned. This may be due to the use of small-diameter contrast catheters and the fact that the ablation catheters were not ablated, which could have led to spontaneous hemostasis.

Anatomically, the anterior region of the superior pulmonary vein is in close proximity to the reflection of the serous pericardium. Therefore, depending on the relationship between the cardiac perforation and the transverse or oblique pericardial sinus, bleeding could occur in the mediastinum if the cardiac perforation is outside the pericardium, or lead to a pericardial tamponade if it is inside the pericardium. Although the exact anatomical relationship between the puncture site and these structures was unclear in the present cases, it is possible that this relationship influenced the amount of bleeding and its hemodynamic impact.

Although several mechanical complications were observed in the present cohort, none occurred during the pulmonary vein isolation procedure itself, and no complications were directly associated with catheter ablation. Nonetheless, the potential risk of LAD-related complications during pulmonary vein isolation cannot be disregarded. These findings highlight the importance of preprocedural identification of the presence and anatomical location of the LAD, as well as careful catheter manipulation in its vicinity to minimize mechanical injury. While it remains challenging to propose specific preventive strategies at this stage, future reports of LAD-related complications during ablation should be thoroughly investigated to establish robust and effective approaches to enhance procedural safety. If an LAD is identified, selecting pulse field ablation or cryoballoon ablation, which can be delivered using soft guidewires or electrode catheters, may help avoid catheter malposition or misplacement. Alternatively, when performing radiofrequency ablation, devising an appropriate isolation line to minimize the risk of mechanical complications may also be an effective strategy. Moreover, recent clinical practice has seen increasing use of high-power, short-duration (HPSD) and very high-power, short-duration (vHPSD) ablation techniques for atrial fibrillation, with accumulating evidence supporting their safety and efficacy [17]. Since lesion depth is known to vary depending on power output and ablation duration [18,19,20,21,22], particular caution should be exercised when ablating near an LAD, whose wall thickness is significantly thinner than that of the surrounding atrial myocardium.

## 5. Limitation

This study has several limitations.

First, as a retrospective observational study, it is subject to potential selection bias and incomplete data acquisition. For example, patients were excluded if they had suboptimal CT image quality, if three-dimensional reconstruction was not feasible, or if contrast agent administration was contraindicated, which may have influenced the detection rate of LADs. Moreover, the absence of strict exclusion criteria (e.g., prior left atrial surgery) may contribute to heterogeneity. However, efforts were made to minimize these effects by excluding non-diagnostic images and reviewing clinical histories to identify prior interventions.

Second, this study was conducted at a single center and involved a relatively homogeneous Asian population, which may limit the generalizability of the findings. Nonetheless, previous studies suggest that LADs may be more prevalent in Asian populations, and given the low overall incidence of mechanical complications during catheter ablation, this population may offer advantages for evaluating such rare associations. Additionally, anatomical differences, such as smaller cardiac chamber sizes among Asians, may have influenced the detection and clinical relevance of LADs.

Third, although LADs were defined as structures protruding outward from the plane of the left atrial wall, there are inherent limitations to the diagnostic precision of this definition. Some morphologically similar structures, such as accessory atrial appendages or left atrial aneurysms, may mimic LADs on imaging. Despite our efforts to differentiate these based on anatomical features, location, and lack of pulmonary vein communication, a degree of misclassification cannot be entirely excluded.

Finally, there are limitations related to the statistical methods employed in this study. The incidence of mechanical complications was exceedingly low, and all such events occurred in the LAD group. Although Firth’s penalized logistic regression was applied to mitigate bias arising from rare event data, the resulting odds ratios were accompanied by wide 95% confidence intervals, reflecting the limited precision of the estimates. Nevertheless, the directionality of the association between LAD and mechanical complications remained consistent with the results of Fisher’s exact test, and a statistically significant relationship was observed. These findings should be interpreted with caution, and confirmation through larger-scale studies is warranted.

Further large-scale prospective investigations are warranted to explore in greater depth the relationship between the presence of an LAD and the incidence of perioperative complications associated with catheter ablation.

## 6. Conclusions

A left atrial diverticulum (LAD) is a more common anatomical structure than generally recognized and may be associated with mechanical complications during AF ablation. Accurate identification of LADs on preoperative CT imaging is essential for anticipating potential procedural risks. In particular, since the anterior region of the right superior pulmonary vein is a frequent site of LAD formation and a common target for ablation, careful catheter manipulation in this area is crucial to minimize complications.

## Figures and Tables

**Table 1:** Patient characteristics.

	LAD (−) *N* = 385	LAD (+) *N* = 210	*p*-Value
Age (SD)	65.6 (10.3)	64.2 (10.2)	0.13
Sex-Female (%)	73 (19.0)	31 (14.8)	0.23
Body mass index (kg/cm^2^) (SD)	24.4 (3.7)	24.6 (4.0)	0.57
Target arrhythmia (%)			
Paroxysmal AF	187 (48.6)	94 (44.8)	0.38
Persistent AF	98 (25.5)	54 (25.7)	
Recurrent AF	71 (18.4)	36 (17.1)	
Atrial flutter	14 (3.6)	11 (5.2)	
Atrial tachycardia	15 (3.9)	15 (7.1)	
CHADS_2_ score (SD)	0.7 (0.9)	0.8 (1.0)	0.74
CHA_2_DS_2_-VASc score (SD)	0.9 (1.2)	0.9 (1.2)	0.78
Hypertension (%)	113 (29.4)	73 (34.8)	0.41
Diabetes (%)	40 (10.4)	21 (10.0)	0.79
Prior stroke (%)	15 (3.9)	7 (3.3)	1
**Blood test**			
Hemoglobin (g/dL) [IQR]	14.4 [13.8, 15.3]	14.5 [13.3, 15.6]	0.96
Creatinine (mg/dL) [IQR]	0.92 [0.79, 1.04]	0.90 [0.79, 1.04]	0.89
Brain natriuretic peptide (pg/mL) [IQR]	82.9 [33.1, 158.5]	67.3 [38.0, 157.1]	0.99
**Echocardiography**			
Left ventricular end-diastolic diameter (cm) [IQR]	4.7 [4.4, 5.2]	4.7 [4.3, 5.1]	0.50
Left ventricular ejection fraction (%) [IQR]	61.2 [56.1, 65.0]	62.1 [55.4, 65.3]	0.80
Left atrium diameter (cm) [IQR]	4.0 [3.6, 4.5]	4.1 [3.7, 4.6]	0.21
Transmitral peak E-wave velocity (cm/s) [IQR]	72.0 [61.0, 85.0]	80.0 [66.0, 88.0]	0.02 *
Transmitral peak A-wave velocity (cm/s) [IQR]	61.0 [49.3, 73.0]	59.0 [47.0, 71.8]	0.52
Transmitral early filling wave deceleration time (ms) [IQR]	187.0 [155.0, 222.0]	179.0 [154.0, 210.0]	0.32
E/e’(IVS) [IQR]	9.1 [0.4, 33.2]	10.4 [4.7, 33.2]	0.01 *

* *p* < 0.05. AF, atrial fibrillation; LAD, left atrial diverticulum.

**Table 2:** Complications during AF catheter ablation.

Complications (%)	LAD (−)	LAD (+)	*p*-Value
Mechanical complication	0 (0.0)	4 (1.9)	0.02
Cerebral infarction	1 (0.5)	0 (0.0)	NA
Myocardial infarction	0 (0.0)	1 (0.7)	NA
Phrenic nerve injury	3 (1.4)	1 (0.7)	NA
Esophageal ulcer	1 (0.5)	0 (0.0)	NA

LAD, left atrial diverticulum.

**Table 3:** Association between mechanical complications and baseline characteristics.

	Univariate Analysis			Multivariate Analysis		
Variables	Odds Ratio	95% CI	*p*-Value	Odds Ratio	95% CI	*p*-Value
Age (per 1 y)	1.03	0.94–1.17	0.58	1.04	0.94–1.18	0.44
Sex-Female	0.52	0–4.91	0.63			
Body mass index (per 1 kg/cm^2^)	1.01	0.75–1.26	0.92			
CHADS_2_ score (per 1 point)	0.71	0.1–1.94	0.59			
CHA_2_DS_2_-VASc score (per 1 point)	0.62	0.08–1.53	0.38			
Hypertension	0.94	0.09–5.75	0.95			
Diabetes	0.96	0.01–9.14	0.98			
Prior stroke	2.81	0.02–27.63	0.55			
Hemoglobin (per 1 g/dL)	1.03	0.98–1.05	0.17			
Creatinine (per 1 mg/dL)	1.24	0.86–1.49	0.15			
Brain natriuretic peptide (per 1 pg/mL)	1	1–1	0.16			
Left ventricular end-diastolic diameter (per 1 cm)	1.06	1–1.13	0.06			
Left ventricular ejection fraction (per 1%)	1.01	0.99–1.01	0.21			
Left atrium diameter (per 1 cm)	1.07	1–1.15	0.05			
Trans-mitral peak E-wave velocity (per 1 cm/s)	0.98	0.89–1.08	0.77			
E/e’(IVS) (per 1)	1.08	0.44–1.31	0.70			
LAD (+)	16.8	1.78–2230.52	0.01 *	17.82	1.88–2369.95	<0.01 *

* *p* < 0.05. LAD, left atrial diverticulum.

## Data Availability

The datasets used in this study are not publicly available due to ethical and privacy concerns, but may be considered for sharing upon request.
